# Antimicrobial Stewardship and Infection Prevention and Control in the Veneto Region, Northeastern Italy: Governance Models, Resources, and Key Challenges Across Hospital and Community Settings—Findings from the ARCO Project

**DOI:** 10.3390/microorganisms13020405

**Published:** 2025-02-13

**Authors:** Paola Anello, Stefano Vianello, Vincenzo Baldo, Enrica Frasson, Umberto Gallo, Roberta Rampazzo, Milvia Marchiori, Mara Carraro, Sara Marchiori, Marta Pigozzo, Vinicio Manfrin, Annarita Mazzariol, Paola De Ambrosis, Davide Gentili, Michele Tonon, Romina Cazzaro, Giovanna Scroccaro, Francesca Russo, Daniele Mengato

**Affiliations:** 1 Hospital Medical Management, ULSS 2 Marca Trevigiana Health Authority, St. Valentino Hospital, Via Palmiro Togliatti, 1, 31044 Montebelluna Treviso, Italy; paola.ring@gmail.com (P.A.); enrica.frasson@studenti.unipd.it (E.F.); 2 Directorate of Health and Social Services, ULSS 6 Euganea Health Authority, Via Enrico degli Scrovegni, 12, 35131 Padova, Italy; stefano.vianello@aulss6.veneto.it; 3 Department of Cardiac Thoracic and Vascular Sciences and Public Health, University of Padua, Via Giustiniani, 2, 35128 Padova, Italy; vincenzo.baldo@unipd.it; 4 Primary Care Pharmaceutical Departments, ULSS 6 Euganea Health Authority, Via Enrico degli Scrovegni, 12, 35131 Padova, Italy; umberto.gallo@aulss6.veneto.it; 5 Hospital Pharmacy, ULSS 5 Polesana Health Authority, St. Maria della Misericordia Hospital, Viale tre martiri, 89, 45100 Rovigo, Italy; roberta.rampazzo@aulss5.veneto.it; 6 Hospital Medical Management, ULSS 6 Euganea Health Authority, Camposampiero Hospital, Via G. Ponzian, 1, 35012 Camposampiero, Italy; milvia.marchiori@aulss6.veneto.it (M.M.); mara.carraro@aulss6.veneto.it (M.C.); 7 Integrated Home Care Service, ULSS 3 Serenissima Health Authority, Via Don Federico Tosatto, 49, 30174 Venezia, Italy; marchiori.sara@aulss3.veneto.it; 8 Hospital Medical Management, ULSS 3 Serenissima Health Authority, “Dell’Angelo” Hospital, Via Paccagnella, 11, 30174 Chirignago-Zelarino, Italy; marta.pigozzo@aulss3.veneto.it; 9 Department of Infectious Diseases, St. Bortolo Hospital, Viale Ferdinando Rodolfi 37, 36100 Vicenza, Italy; vinicio.manfrin@aulss8.veneto.it; 10 Department of Diagnostics and Public Health, University of Verona, Strada le Grazie, 8, 37134 Verona, Italy; annarita.mazzariol@univr.it; 11 Regional Directorate for Pharmaceutical and Medical Devices, Rio Novo-Dorsoduro 3493, 30123 Venezia, Italy; paola.deambrosis@regione.veneto.it (P.D.A.); giovanna.scroccaro@regione.veneto.it (G.S.); 12 Regional Directorate for Prevention, Food Safety, Veterinary, Public Health-Veneto Region, Rio Novo-Dorsoduro 3493, 30123 Venezia, Italy; davide.gentili@regione.veneto.it (D.G.); michele.tonon@regione.veneto.it (M.T.); francesca.russo@regione.veneto.it (F.R.); 13 Regional Directorate for Health Programming, Rio Novo-Dorsoduro 3493, 30123 Venezia, Italy; romina.cazzaro@regione.veneto.it; 14 Hospital Pharmacy Department, Azienda Ospedale-Università Padova, Via Nicolò Giustiniani, 2, 35128 Padova, Italy

**Keywords:** antimicrobial stewardship, infection prevention and control, public health, healthcare governance, community healthcare, multidisciplinary approach

## Abstract

Antimicrobial resistance represents a global health challenge, with Italy among the most affected countries in Europe. In response, the Veneto Region developed governance models to enhance antimicrobial stewardship (AMS) and infection prevention in both hospital and community settings. This study assessed the implementation of these models and explored strategies for improvement. A survey targeting hospital medical and district health management, hospital pharmacies, and primary care pharmaceutical departments was conducted to evaluate governance adherence, resource allocation, training, and reporting practices. Findings were analyzed by a focus group comprising regional experts, infectious disease specialists, microbiologists, and policymakers, which identified critical gaps and proposed actionable recommendations. Results revealed disparities in AMS implementation between hospital and community care, with key challenges including limited multidisciplinary collaboration, inconsistent resource distribution, and gaps in training. The focus group emphasized the need for stronger governance, standardized protocols, and improved communication to address these shortcomings. This study underscores significant gaps and opportunities within the Veneto Region’s healthcare system and provides a framework for enhancing AMS and infection prevention strategies, bridging the divide between hospital and community care to combat antimicrobial resistance effectively.

## 1. Introduction

Antimicrobial resistance (AMR) is a global health threat with alarming projections for the future [1,2]. By 2050, it is estimated that infections caused by antibiotic-resistant bacteria will surpass cancer as the leading cause of death worldwide [3]. Italy, in particular, faces some of the highest levels of resistance for critical pathogens like *Klebsiella pneumoniae* and *Escherichia coli*. The country bears one of the heaviest burdens of AMR in Europe, with over 10,000 deaths annually attributed to resistant infections—accounting for more than a third of all AMR-related deaths in the EU. Developing robust antimicrobial stewardship (AMS) programs in hospitals is critical to addressing this issue [4]. However, it is equally important to focus on primary care settings, where 90% of antibiotic prescriptions are made, with two-thirds originating from primary care physicians. This makes primary care a key area for AMS initiatives aimed at optimizing antibiotic use and preventing the spread of resistance [5].

National Action Plans (NAPs) for AMR can serve as a crucial tool in shaping national strategies to combat this growing threat [6]. These plans provide a structured framework for addressing AMR across various sectors, promoting collaboration between healthcare, veterinary, and environmental systems. However, as highlighted in various studies, the existence of NAPs alone is not sufficient to ensure success [7]. In Italy, the first national plan was introduced with the National Action Plan on Antimicrobial Resistance 2017–2020 (updated in 2022), along with its related surveillance system, SPiNCAR (Surveillance and Prevention of Healthcare-Associated Infections and Antimicrobial Resistance), aimed at monitoring the implementation of the NAP [8,9,10].

The Italian National Health Service is a decentralized public system with three levels: central, regional, and local. While the national government sets overall health policies and standards, the 21 regions and autonomous provinces have significant autonomy in managing healthcare services. Each region is governed by elected officials who oversee healthcare delivery through hospital trusts and Local Health Authorities (LHAs). LHAs are responsible for providing healthcare within a defined geographic area. They are managed by General Managers appointed at the regional level and are further divided into Local Health Units (LHUs), which integrate hospital and community-based services. Major hospitals often operate as semi-independent public entities known as ‘public hospital enterprises’ or specialized hospitals [11]. In the Veneto region, north-east of Italy, there are nine LHAs and three specialized hospitals. The nine LHAs vary in size, with populations ranging from 300,000 to nearly 1 million. The community area of each LHA is divided into one or more territorial areas, each with its own director [12]. Primary care within LHAs is provided by a network of general practitioners who act as gatekeepers to higher levels of care. Home care services, which are less complex than those provided in hospitals, are delivered either at the patient’s home or in nursing homes. The Community Hospital is a healthcare facility with 20 beds per 100,000 inhabitants, designed for patients requiring short-term, low-intensity care [13].

**Figure 1:**
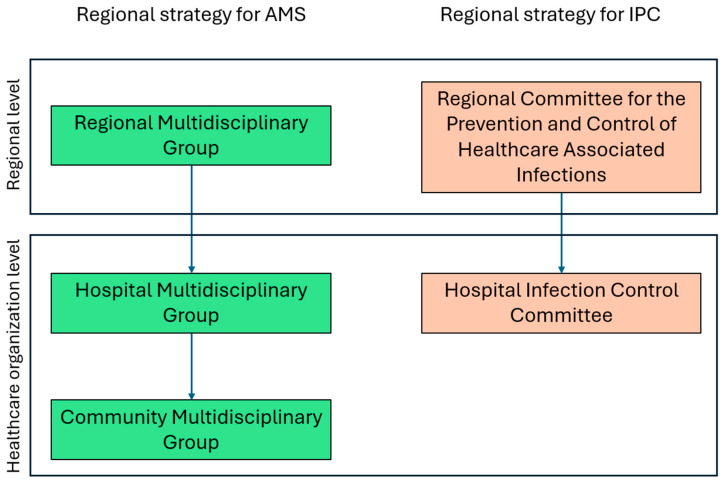
Veneto region’s strategy for AMS and IPC.

In the Veneto Region, the first NAP was adopted in 2019 through a regional decree, which outlined strategies for the proper use of antibiotics in human health and introduced a regional plan for the surveillance, prevention, and control of healthcare-associated infections (HAIs). The Veneto Region’s strategy for antimicrobial use includes a comprehensive governance structure for AMS, divided into three levels: the Regional Multidisciplinary Group at the regional level, the Hospital Multidisciplinary Group at the hub hospitals, and the Community Multidisciplinary Group in territorial health organizations, managing AMS activities at community level [14]. Additionally, the governance model for Infection Prevention and Control (IPC) mandates the establishment of the Hospital Infection Control Committee, chaired by the hospital medical director, to oversee infection control measures. Additionally, a Regional Coordinating Committee ensures alignment and consistency across all healthcare settings, both hospital-based and community-based (Figure 1) [15]. It guarantees consistency by developing standardized protocols and guidelines for AMS and IPC, which provide a framework for uniform practices. To foster collaboration and continuous improvement, the committee facilitates knowledge sharing through regular meetings and educational events, enabling the exchange of best practices among healthcare professionals. Furthermore, the committee tracks the progress of AMS and IPC implementation through specific key performance indicators (KPIs), ensuring that the goals of these programs are met and sustained over time. The committee is also responsible for monitoring the implementation and adherence to the regional plan, ensuring that AMS and IPC initiatives are effectively carried out.

The plan for the surveillance, prevention, and control of HAIs also introduced the role of Link Professionals, healthcare professionals (e.g., nurses, technicians, doctors, etc.) who play a leadership role within their facility on infection risk-related matters [16].

This paper presents the findings of the ARCO project (Approcci di Rete per il Contrasto all’Antimicrobico Resistenza Ospedale-Territorio—Network Approaches for Combating Antimicrobial Resistance between Hospital and Community), developed through collaboration among three scientific societies: ANMDO (National Association of Hospital Medical Management), SIFACT (Italian Society of Clinical Pharmacy and Therapeutics), and CARD (Confederation of Regional District Associations). The ARCO working group includes a multidisciplinary team from various healthcare organizations in the Veneto Region, comprising hospital medical directors, hospital pharmacists, infection risk specialists, primary care pharmacists, and coordinators in integrated home care and district functions. The primary objective of the ARCO project is to evaluate the implementation of regional governance models for IPC and AMS across healthcare organizations in the Veneto Region. Additionally, the project involves analyzing data from a focus group of key stakeholders and experts to gain a deeper understanding of current AMS and IPC practices. This analysis will help identify best practices and areas for improvement, contributing to the refinement of AMS and IPC strategies within the region.

## 2. Materials and Methods

### 2.1. Survey Design and Distribution

In May 2023, a subgroup of the ARCO working group developed a draft questionnaire to evaluate adherence to regional governance models for IPC and AMS and to explore the integration between hospital and community care settings in the Veneto region.

Four tailored questionnaires were designed, targeting Hospital Medical Management (HMM), District Health Management (DHM), Hospital Pharmacies (HP), and Primary Care Pharmaceutical Departments (PCPD). A literature search was conducted on published standards and regional directives for IPC and AMS to inform the questionnaire design. The questions included topics such as staffing levels for AMS-related activities (pharmacists, nurses, and physicians), the existence and functioning of AMS programs, training on AMR and AMS, and reporting practices related to antibiotic consumption, protocol adherence, and surveillance data. The survey also assessed the implementation of governance models and the level of coordination between hospital and community settings.

A web-based survey tool (Google Forms, Google Inc., Mountain View, CA, USA) was utilized. The initial draft survey was distributed in July 2023 to all members of the ARCO working group to test the readability and clarity of the questions. Based on their feedback, adjustments were made to improve the structure and ensure alignment with the study objectives.

The final surveys were distributed to LHAs with instructions to forward them to the appropriate targets (HMM, DHM, HP, and PCPD). An invitation letter was sent to LHAs by the Regional Directorate of Pharmaceutical Services and Medical Devices and the Directorate of Prevention and Food Safety. The survey was open from 3 November to 11 November 2023, and responses were completed by representatives from HMM, DHM, HP, and PCPD.

Data analysis was conducted using Microsoft Excel (Microsoft Office 365, Microsoft Corporation, Redmond, WA, USA) and Stata (version 12.0, StataCorp, College Station, TX, USA). The Shapiro–Wilk normality test was applied to assess the distribution of quantitative variables, all of which were found to be non-parametric. Frequencies, percentages, medians, and interquartile ranges (IQR) were calculated for each variable.

Comparative analyses were performed to evaluate differences in variable distributions across categories, such as hospital versus community settings for the identification, training, and assigned objectives of LP, as well as hub versus other hospitals for the availability of infectious disease consultation services. The hub category included university and provincial hub hospitals, while the other hospitals category comprised spoke hospitals and one highly specialized oncological hospital. The χ^2^ test was used for categorical variables, and the Wilcoxon Rank Sum Test was applied for continuous variables due to their non-parametric distribution.

### 2.2. Focus Group

Following the analysis of survey data, a structured focus group was conducted on 10 May 2024 to provide additional qualitative insights and contextualize the findings from the survey. The focus group aimed to address key issues in IPC and AMS practices, leveraging multidisciplinary expertise to identify gaps and develop actionable recommendations.

The focus group involved 18 participants, including members of the ARCO working group, two representatives from the regional directorates, and a panel of experts. The expert panel comprised infectious disease specialists, a microbiologist, a hospital hygienist, pharmacists from hospital and territorial settings, representatives from integrated home care services, and a medical writer. This diverse group was chosen to ensure a wide range of perspectives on IPC and AMS challenges across healthcare settings.

The focus group was structured into three thematic sessions, each lasting approximately one hour, with clear objectives and predefined discussion prompts. The sessions were moderated by two facilitators of the ARCO working group, with expertise in IPC, AMS, and healthcare management, to ensure balanced participation and focused discussions.

Thematic areas of focus included:

- Professional Commitment: Discussion of staffing, roles, and AMS committee structures;
- Activities and Integration: Exploration of inter-organizational coordination and differences in IPC practices between hospital and community settings;
- Audit, Feedback, and Reporting: Analysis of data interpretation, reporting mechanisms, and feedback strategies.

Moderators guided the discussions using predefined prompts aligned with each thematic session. The medical writer documented the proceedings, distilling key insights and actionable recommendations that emerged during the discussions. These proceedings were subsequently thematically analyzed by the ARCO working group to identify recurring patterns, challenges, and opportunities. This thorough analysis ensured a nuanced understanding of the issues discussed and served as the foundation for developing targeted interventions and practical recommendations to enhance AMS and IPC programs across diverse healthcare settings.

## 3. Results

The survey was completed by healthcare professionals, starting with 21 HMMs that represented 34 hospital facilities. These included 26 spoke hospitals, 2 highly specialized hospitals, 4 hub hospitals, and 2 university hospitals, accounting for a total of 12,115 beds. This response represented 100% of the healthcare organizations in the region. Additionally, 22 DHM participated, covering 22 healthcare districts. These districts provide healthcare services to a total of 4,133,010 residents out of a total population of 4,906,000, also representing 100% of the region’s healthcare organizations. Among HP, 16 out of a total of 19 participated, representing 100% of the healthcare organizations in the region. Lastly, 8 PCPDs out of a total of 9 responded, representing 89% of the community pharmacies and healthcare organizations in the region, with one community pharmacy per healthcare organization.

### 3.1. Governance and Organizational Structure

According to HMM, three out of four healthcare organizations reported having a formalized annual plan for IPC. Additionally, 1 out of 2 organizations indicated having a formalized antimicrobial stewardship plan in place.

**Figure 2:**
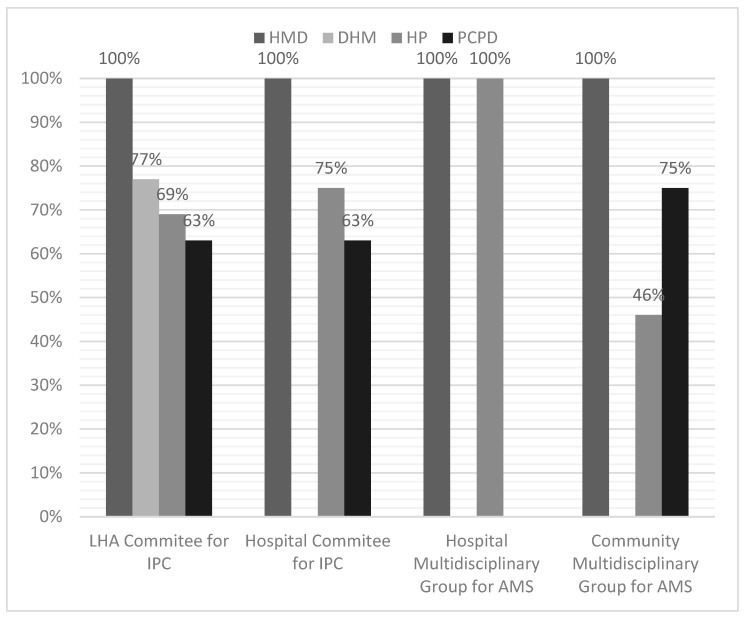
Establishment of Corporate Committees according to Hospital Medical Management (HMM), District Health Management (DHM), Hospital Pharmacies (HP), and Primary Care Pharmaceutical Department (PCPD). LHA: Local Health Authority.

Each respondent could report his or her knowledge of the activation of various company or local committees active in the area of AMS and IPC. These include the LHA Committee for IPC, the Hospital-based Committee for IPC, the Hospital Multidisciplinary Group for AMS, and the Community Multidisciplinary Group for AMS. All HMM reported that the required committees had been formally established, whereas the other corporate divisions did not provide the same response (Figure 2), even within the same healthcare organization.

### 3.2. Human Resources and Link Professional for AMS and IPC

Table 1 presents the allocation of human resources (nurses, physicians, and pharmacists) dedicated to IPC and AMS programs across hospital and community care settings. In hospitals, there were 33 fully dedicated infection control nurses (0.25 per 100 beds; IQR 0.18–0.32) and 12 partially dedicated nurses (0.00 per 100 beds; IQR 0.18–0.32). Six public health specialists were fully dedicated (0.00 per 100 beds; IQR 0.0–0.18), while 28 were partially dedicated (0.29 per 100 beds; IQR 0.11–0.50). Hospital pharmacists had only one fully dedicated professional, with 33 partially dedicated pharmacists (0.35 per 100 beds; IQR 0.17–0.84).

In community settings, no pharmacists were fully dedicated, though 12 were partially involved (0.004 per 1000 inhabitants; IQR 0.002–0.005). In primary care, no nurses or physicians were fully dedicated to AMS/IPC programs, but 21 nurses (0.005 per 1000 inhabitants; IQR 0.000–0.009) and 33 physicians (0.006 per 1000 inhabitants; IQR 0.004–0.013) were partially involved. Similarly, in community hospitals, no fully dedicated staff were reported, but 41 nurses (3.41 per 100 beds; IQR 0.00–5.30) and 22 physicians (0.08 per 100 beds; IQR 0.00–0.14) were partially involved. In domiciliary care, no fully dedicated staff were present, but 12 nurses (0.002 per 1000 inhabitants; IQR 0.000–0.009) and 18 physicians (0.000 per 1000 inhabitants; IQR 0.000–0.005) were partially involved in IPC/AMS activities.

Table 2 provides data on the identification, training, and involvement of Link Professionals in IPC and AMS programs across both hospital and community settings. In hospitals, 76% of link professionals are formally identified, whereas only 36% are formally identified in community settings, with 50% of link professionals in the community not identified at all. Differences between hospital and community settings are statistically significant (*p* < 0.001).

Regarding training, 33% of hospital-based link professionals have received dedicated IPC training, while 48% have participated in non-specific programs, and 19% have not undergone any training. In community settings, these figures are lower: 18% have received dedicated training, 73% have attended non-specific programs, and 9% have not received any training. Link professionals are involved across a range of services. In hospitals, they are present in inpatient wards (100%), emergency departments (86%), operating theaters (81%), and diagnostic imaging services (71%). They also contribute to outpatient services (67%) and daycare activities (48%). In community settings, link professionals are primarily engaged in primary care (91%), integrated home care (55%), intermediate care facilities (36%), and residential facilities (36%). The objectives assigned to link professionals vary significantly between hospital and community settings. In hospitals, 76% are involved in training and promoting best practices, 62% participate in HAI reporting, and 52% utilize checklists to ensure IPC measures are followed. In community settings, similar activities are observed, with 82% participating in HAI reporting and 55% focusing on best practices promotion. However, the formalization of these objectives remains limited, with only 10% of hospital-based and 9% of community-based link professionals having formalized, monitored objectives with shared outcomes.

### 3.3. Infectious Disease (ID) Consultation Services

The survey results indicate that 75% of healthcare organizations have an infectious disease ward, typically located in hub hospitals. Additionally, 3 out of 12 organizations have established agreements with other institutions to provide infectious disease consultations, while three have hired infectious disease specialists on a freelance basis. Five organizations reported employing infectious disease specialists in departments outside of the infectious diseases unit, ranging from one to three professionals. These specialists are employed in diverse areas, including two in medical management, two in geriatrics, one in internal medicine, and one in pediatrics. This highlights the varied organizational approaches to securing infectious disease expertise across healthcare settings.

As shown in Table 3, there is significant variability in the availability of ID consultation services between hub hospitals and other facilities. No hub hospitals reported a lack of ID consultation services, whereas 6.7% of other hospitals had no availability. While 83.3% of hub hospitals provide ID consultation services 6 to 7 days per week, only 20.0% of other hospitals achieve this level of access. Conversely, 40.0% of other hospitals offer services only 1 to 3 days per week, a level not observed in any hub hospitals.

Regarding hours per week, none of the hub hospitals provide less than 20 h of ID consultation, compared to 66.7% of other hospitals. Additionally, 50.0% of hub hospitals provide full-time consultation (168 h per week), a service unavailable in any of the other hospitals.

Hub hospitals provide more comprehensive access, with a median of 7 days per week (IQR 6–7), compared to 4 days per week (IQR 1–5) in other hospitals (*p* = 0.008). Similarly, in terms of hours per week, hub hospitals have a median of 109 h (IQR 40–168), whereas other hospitals provide a median of only 14 h (IQR 4–30) (*p* = 0.003).

### 3.4. Analysis of Antibiotic Consumption Reports and Indicators in Hospitals and Primary Care Settings

The analysis highlights several key points in the reporting of antibiotic consumption across hospital and primary care settings (Table 4). In hospital reports, almost all (94%) included economic indicators such as expenditure for ATC category J01, while 63% included quantitative indicators measuring consumption in dosage units or pieces. Notably, 75% of hospital reports also incorporated quantitative indicators expressed in Defined Daily Dose (DDD) and qualitative indicators through the AWaRe classification. However, only 25% of the reports included ESAC indicators, showing limited attention to the European Surveillance of Antimicrobial Consumption.

In comparison to hospitals, primary care reports demonstrated a lower use of quantitative indicators expressed in DDD (38% vs. 75%). However, there was a significant reliance on indicators that measure the number of patients treated, observed in 50% of primary care reports. Similarly, the qualitative AWaRe classification was present in 63% of primary care reports compared to 75% in hospitals, while none of the primary care reports included ESAC indicators.

In terms of infection management guidelines, 38% of primary care reports addressed upper respiratory, lower respiratory, and lower urinary tract infections, with a smaller percentage (13%) covering soft tissue infections.

### 3.5. Focus Group Recommendations

The focus group provided a series of detailed recommendations aimed at improving AMS and IPC programs across various healthcare settings. These recommendations, summarized in Table 5, cover key areas such as governance, human resources, link professionals, infectious disease consultation services, and antibiotic consumption reporting in hospitals and primary care. The group emphasized the importance of tailoring AMS and IPC objectives to the specific needs of healthcare organizations, ensuring these goals are incorporated into senior leadership performance evaluations. Additionally, there was a strong emphasis on improving resource allocation, providing standardized training, and formally recognizing link professionals, especially in community settings where these roles are underdeveloped. The focus group also highlighted the need to expand infectious disease consultation services in both spoke hospitals and community care settings to provide continuous support for AMS activities rather than relying on ad hoc consultations. These recommendations aim to address existing gaps in resources and governance structures, ensuring a more cohesive and effective approach to AMS and IPC implementation.

## 4. Discussion

National action plans are useful but not sufficient for the effective implementation of AMS and IPC measures [6]. The aim of our project was to collect data through surveys and, following a focus group discussion, to identify best practices and approaches within HCAs that could be integrated into regional and local plans.

The ARCO project gathered expert recommendations to strengthen governance within HCAs and enhance leadership commitment by defining clear objectives and allocating resources to incentivize AMS program implementation. Broom et al. highlighted the challenge of prioritizing long-term public health goals, such as AMR, over short-term performance-driven objectives, underscoring the need for clearly defined, long-term objectives aligned with AMS goals to ensure accountability and sustained leadership commitment [17]. Similarly, Jeleff et al. emphasized that AMS compliance must go beyond formal documentation to include meaningful resource allocation and concrete actions that drive real improvements [18]. Further recommendations focused on strengthening local and regional governance. Regional and local AMR committees can tailor interventions to local needs, better-aligning efforts with regional epidemiological contexts. These committees foster collaboration across hospitals and community settings, enabling quicker responses to resistance trends [7,19]. However, localized approaches also present challenges, such as the risk of fragmentation and duplicated efforts due to overlapping roles [17,20,21]. To address this, minimum operational standards should be established, including regional toolkits accessible to healthcare professionals, modeled after the AMS Toolkit TARGET and Start Smart Then Focus from the UK NHS [22]. Veneto could strengthen its program by integrating key elements of these UK initiatives, such as adapting guidelines to local resistance patterns, creating tailored patient education campaigns, and implementing standardized training for healthcare providers. Additionally, mandatory audits, feedback systems, and 48–72 h antibiotic reviews in hospitals would facilitate continuous assessment and optimization of therapy. These measures, combined with implementation-focused content and specific indicators for organizational plans, would help ensure consistent standards across all settings.

Additionally, investment in communication and transparency is crucial, ensuring that all healthcare professionals are informed about existing committees and medium- to long-term plans, with key information made accessible via the organization’s website. Furthermore, sensitization campaigns targeting the general population should be prioritized [23]. These will include mass media outreach through television, radio, and social media platforms to disseminate evidence-based messages about antimicrobial resistance and appropriate antibiotic use. Additionally, community engagement initiatives, such as educational events in schools, workplaces, and community centers, will foster grassroots awareness. Tailored patient-centered materials, including brochures, posters, and infographics, will be distributed across healthcare facilities and pharmacies to ensure accessibility to diverse populations.

Our work focuses on the human resources necessary for effective AMS and IPC programs, highlighting the critical importance of adequate staffing for their success and sustainability. In hospitals, we observed a median of 0.25 IPC nurses per 100 beds (IQR 0.18–0.32) and 0.00 IPC physicians per 100 beds (IQR 0.00–0.18) fully dedicated to IPC, which aligns with the national ratio for nurses but falls short of European data, where the average is 0.80 IPC nurses and 0.15 physicians per 100 beds [24,25]. Italian guidelines from 1980 recommend 0.25–0.4 IPC nurses and 0.1 physicians per 100 beds, while the European Centre for Disease Prevention and Control has recently updated its guidelines to recommend 1 IPC nurse per 100 beds, reflecting the increasing need for specialized personnel [26,27]. Where national standards are lacking—such as in HP, PCPD, and community healthcare—dedicated personnel are often absent. Our study found a median of 0.00 HP per 100 beds, significantly lower than the European average of 0.1221. Several guidelines suggest that a ratio of 1 HP per 100 beds is necessary for effective clinical pharmacy services, particularly in AMS programs [28,29,30]. While hospital standards and resources are frequently studied, there is a significant gap in research and documentation on human resources in community healthcare settings in Italy. This lack of data underscores the urgent need for the definition of clear standards and the subsequent mapping of available resources. Addressing this issue is essential to ensure consistency in AMS and IPC implementation, bridging the gap between hospitals and community care settings.

Expanding services and appointing permanent AMS reference figures in spoke hospitals and primary care is essential to integrate AMS into routine clinical practice, shifting from ad hoc consultations to continuous support and improving antimicrobial management.

The ID consultation services, particularly in spoke hospitals and community settings, pose significant barriers to effective AMS and IPC implementation. According to our study, hub hospitals consistently demonstrate superior access, offering a median of 7 days (IQR 6–7) and 109 h (IQR 40–168) of ID consultations weekly, compared to only 4 days (IQR 1–5) and 14 h (IQR 4–30) in other hospitals. These disparities exacerbate challenges in maintaining consistent AMS practices across healthcare settings. Notably, 66.7% of other hospitals provide fewer than 20 h of ID consultations weekly, with no full-time coverage, underscoring a critical gap in service provision.

Telemedicine and teleconsultation services present a viable and cost-effective solution to these disparities. Evidence supports their role in reducing antibiotic misuse, patient length of stay, and unnecessary hospital transfers, particularly in underserved areas [31,32,33]. For instance, Vento et al. demonstrated the success of integrated telehealth programs in improving AMS outcomes across multiple small community hospitals by offering round-the-clock ID consultations and tailored ASP support [34]. Similarly, Vestesson et al. highlighted the potential of telemedicine to enhance diagnostic accuracy and adherence to AMS protocols, mitigating the absence of on-site expertise [35]. These findings emphasize the value of regional investments in telemedicine platforms to bridge existing gaps, ensuring equitable access to AMS expertise while being both cost-effective and self-sustaining [31,32,33].

However, telemedicine alone cannot fully resolve these issues. It is also crucial to establish clear staffing standards for AMS teams to ensure adequate and equitable resources across all healthcare settings. A study from another northern Italian region found that for a population of 312,000 inhabitants, an AMS program required four full-time infectious disease consultants, 0.9 FTE microbiologists, and 0.9 FTE pharmacists, alongside an IPC team [36], underscoring the importance of defining minimum resource requirements to guide the development and implementation of AMS teams in both hospital and community settings. Particularly in the community setting, interdisciplinary collaboration between pharmacists, GPs, and infectious disease specialists can effectively address gaps in AMS implementation. The Australian GPPAS model exemplifies this approach, promoting GP-pharmacist collaboration through education on antimicrobial resistance, diagnostic stewardship, and regular prescription reviews. By integrating shared decision-making and practical tools such as training sessions and real-time communication, the model enhances AMS in primary care, reducing inappropriate antimicrobial use and improving patient outcomes [37]. Implementing similar strategies in regional AMS frameworks could ensure consistent and effective stewardship across community settings [38].

Link Professionals play a pivotal role in the success of IPC programs, serving as the connection between policy directives and their practical implementation [16]. However, as evidenced by our findings, significant gaps persist in the recognition and standardization of this role, particularly in community care settings. To address these barriers, establishing a formal system of recognition and incentives is essential. This should include financial compensation, career progression opportunities, and symbolic recognition to elevate the status of link professionals, creating an annual, structured training curriculum tailored to the specific needs of community settings. This effort should be led by the Regional Coordinating Committee in collaboration with academic institutions and scientific societies and should include key elements such as core modules on AMS and IPC, practical workshops, and case studies addressing setting-specific challenges, along with periodic assessments to evaluate competency. Implementation and monitoring should be overseen by the Regional Directorate for Health Programming in collaboration with other Regional Directorates, ensuring alignment with regional and national goals. Either formalizing the objectives assigned to link professionals or creating a system of regular performance monitoring would further enhance their impact, fostering commitment and accountability across the healthcare organization [16,36]. Moreover, our study also revealed that only 50% of DHM applied the provisions related to linking professionals from Regional Decree 1402/2019 to their context. This finding underscores the need for explicit regional standards tailored to community settings to bridge the gap between hospital and community IPC practices concerning link professionals. Addressing these gaps is crucial for fostering a cohesive and effective IPC framework.

The survey highlights the need for standardized antibiotic consumption reporting across hospitals and primary care. Primary care reports often emphasize economic indicators rather than clinical metrics like DDD or the AWaRe classification. The focus group recommended standardizing reports to include both economic and clinical data for a comprehensive understanding of antibiotic use. Additionally, specific prescribing guidelines for primary care are necessary to reduce inappropriate antibiotic use. The ARCO Project also identified gaps in monitoring and reporting adherence to AMS and IPC protocols in the Veneto Region. Implementing standardized indicators and transparent reporting, like the governance model in England, could improve accountability, enhance performance tracking, and foster a more consistent approach to stewardship across the region [39,40].

The focus group reminded the long-standing problem of the application of the General Data Protection Regulation (GDPR) in Italy, which often represents a barrier to sharing data between health authorities and interconnection of health informational flows [41,42]. Quality of data is fundamental to guarantee efficacious health systems governance and disease prevention and control, especially as regards field epidemiology and, consequently, management of HAI and AMR, since these are issues that distress across different healthcare services and spread without taking into account territorial boundaries [40,43]. Successful international initiatives, such as Austria’s COVID-19 data platform and Finland’s Findata system, demonstrate how anonymization and centralized access can overcome privacy barriers while maintaining compliance with GDPR. Implementing similar models in Italy could enhance data integration, improving health system governance and responses to HAI and AMR challenges [44].

The ARCO project may seem similar to SPiNCAR, the tool used to monitor the implementation of the Italian NAP [10]. However, while SPiNCAR focuses on assessing the application of Italian NAP within HCAs, it does not examine internal organizational models, resource allocation, or regional strategies for managing AMS and IPC. Furthermore, the ARCO project aims to provide a broader perspective by incorporating insights from a wider range of professionals and offering a more detailed analysis of resource allocation and organizational structures. Therefore, results from SPiNCAR and ARCO could be integrated to provide a more comprehensive and multi-faceted approach to governance for AMS and IPC across different levels of the Regional Health Service.

To our knowledge, this is the first study to provide a comprehensive understanding of HCAs’ perspectives and actionable measures by combining survey data with focus group insights to identify best practices and areas for improvement. Previous studies have assessed AMS activities through surveys in community healthcare organizations, interviews with hospital managers, or by examining the perspectives of key experts and stakeholders involved in AMR policy [17,18,20]. However, no previous study has undertaken such a thorough contextual analysis, administering questionnaires across various structures within local healthcare organizations and subsequently gathering expert recommendations on organizational context, along with insights from regional leadership, in a regional audit approach. This project serves as a foundational step that can be advanced through a well-defined implementation timeline and measurable indicators. Short-term priorities include stakeholder engagement and resource allocation, followed by medium-term objectives such as infrastructure development and educational programs. Long-term goals focus on the full implementation and evaluation of the proposed measures. Effectiveness will be tracked using key performance indicators like reductions in antimicrobial consumption and resistance rates, adherence to AMS and IPC protocols, and assessments of professional knowledge. Regular monitoring, annual revisions, and open publication of metrics will ensure transparency and ongoing improvement. The ARCO project approach may be relevant to other Italian regions or other settings facing similar barriers in implementing regional and national directives within the realities of local healthcare organizations through a pragmatic approach. Adopting a regional audit framework and prioritizing interdisciplinary collaboration, as demonstrated in this study, can serve as a practical blueprint for improving AMS outcomes and addressing AMR challenges on a broader scale.

Nevertheless, the study is not without its limitations. A significant challenge is the reliance on self-reported data from healthcare organizations, which could introduce reporting biases [43]. Furthermore, while the study offers valuable insights specific to the Veneto Region, its findings may not be fully generalizable to other regions due to variations in healthcare structures and challenges. Despite these limitations, the study provides actionable insights for optimizing AMS and IPC plans, contributing meaningfully to the broader goal of combating antimicrobial resistance.

In conclusion, while significant progress has been made in establishing governance frameworks for AMS and IPC in the Veneto Region, targeted operational improvements are essential for healthcare units to fully realize the potential of these programs. Strengthening unified governance structures, standardizing antibiotic consumption reports, expanding infectious disease consultation services, and ensuring adequate staffing are critical steps to enhancing the region’s response to antimicrobial resistance. The ARCO project provides a valuable foundation for these efforts, offering actionable recommendations that will be key to driving sustained improvements across both hospital and community care settings.

## Figures and Tables

**Table 1:** Standard of human resources (physicians and nurses) partially and fully dedicated to IPC and AMS programs in hospitals and community care: absolute numbers, medians and interquartile ranges of professionals per bed in each of the examined facilities.

Human Resource for IPC/AMS Programs	Fully Dedicated			Partially Dedicated		
	Sum	Median	IQR	Sum	Median	IQR
Hospital						
IPC nurses (per 100 beds)	33	0.25	0.18–0.32	12	0.00	0.00–0.31
IPC physicians (per 100 beds)	6	0.00	0.00–0.18	28	0.29	0.11–0.50
Hospital pharmacists (per 100 beds)	1	0.00	0.00–0.00	33	0.35	0.17–0.84
Community						
Primary Care Pharmacists (per 1000 inhab)	0	-	-	12	0.004	0.002–0.005
Primary care (per 1000 inhab)						
nurse	0	-	-	21	0.005	0.000–0.009
physicians	0	-	-	33	0.006	0.004–0.013
Community hospital (per 100 beds)						
nurse	0	-	-	41	3.41	0.00–5.30
physicians	0	-	-	22	0.08	0.00–0.14
Domiciliary care (per 1000 inhab)						
nurse	0	-	-	12	0.002	0.000–0.009
physicians	0	-	-	18	0.000	0.000–0.005

**Table 2:** Identification, training, service involvement, and assigned objectives of link professionals in hospital and community settings.

	Hospitals		Community		*p* Value (χ^2^ Test)
	n	Percentage	n	Percentage	
Identification of link professional					
no	0	0%	11	50%	<0.001
not formally identified	5	24%	3	14%	
formally identified	16	76%	8	36%	
Training program of link professional					
not provided	4	19%	1	9%	0.395
not dedicated	10	48%	8	73%	
dedicated	7	33%	2	18%	
Identified services					
Inpatient Wards	21	100%	-	-	n.a.
Emergency Department	18	86%	-	-	
Operating theater	17	81%	-	-	
Diagnostic Imaging	15	71%	-	-	
Outpatient Services	14	67%	-	-	
Day Care Activities	10	48%	-	-	
Immunotransfusion Service	6	29%	-	-	
Analysis Laboratory	6	29%	-	-	
Pathological Anatomy	6	29%	-	-	
Primary Care	-	-	10	91%	
Integrated Home Care	-	-	6	55%	
Intermediate Care Facilities	-	-	4	36%	
Residential Facilities	-	-	4	36%	
District Management	-	-	2	18%	
Territorial Operational Center	-	-	1	9%	
Assigned objectives for link professionals					
Auditing/review of documentation	8	38%	5	45%	0.206
Procedure/bundle development	10	48%	0	0%	
Surveillance data collection	10	48%	2	18%	
Checklist application	11	52%	5	45%	
HAI/adverse event reporting	13	62%	9	82%	
Training/promotion of best practices	16	76%	6	55%	
Objectives for link professionals					
Not assigned	4	19%	-	-	0.144
Assigned, but not formalized	11	52%	4	36%	
Formalized and periodically monitored	4	19%	6	55%	
Formalized, monitored, and results shared	2	10%	1	9%	

**Table 3:** Availability of infectious disease consultation services in hub and other hospitals.

Category	Hub Hospitals		Other Hospitals		*p*-Value (χ^2^ Test)
	n	Percentage	n	Percentage	
Days per Week with ID Consultation					
No availability	0	0.0%	1	6.7%	0.052
1 to 3 days per week	0	0.0%	6	40.0%	
4 to 5 days per week	1	16.7%	5	33.3%	
6 to 7 days per week	5	83.3%	3	20.0%	
Hours per Week with ID Consultation					
0–20 h per week	0	0.0%	10	66.7%	0.008
21–30 h per week	1	16.7%	2	13.3%	
31–70 h per week	2	33.3%	3	20.0%	
Full-time (168 h per week)	3	50.0%	0	0.0%	

**Table 4:** Analysis of antibiotic consumption reports and indicators in hospitals and primary care settings. DDD: Defined Daily Dose; ATC: Anatomical Therapeutic Chemical Classification; PD: patient days; DOT: days of therapy.

	Hospitals		Primary Care	
	n	%	n	%
Reports on antibiotic consumption				
Hospital wards	16	100%	-	-
General practitioners	-	-	6	75%
Community pediatrician	-	-	0	0%
General practitioners + community pediatrician	-	-	2	25%
Indicators				
Total expenditure for ATC category J01	15	94%	3	38%
Treated (number)	4	25%	4	50%
Dosage units/pieces consumed for ATC category J01	10	63%	3	38%
Total DOT	1	6%	0	0%
DOT/100 PDs or 1000 inhab	1	6%		0%
Total DDD	12	75%	3	38%
DDD/100 PDs or 1000 inhab	14	88%		0%
DDD/100 PDs or 1000 inhab for beta-lactams/enzyme inhibitors	9	56%	2	25%
DDD/100 PDs or 1000 inhab for third-generation cephalosporins	10	63%	2	25%
DDD/100 PDs or 1000 inhab for carbapenems	13	81%	1	13%
DDD/100 PDs or 1000 inhab for fluoroquinolones	13	81%	2	25%
Consumption by AWaRE classes	12	75%	5	63%
ESAC Indicators	4	25%	0	0%
Protocols/guidelines for infection management in primary care				
Management of upper respiratory tract infections	-	-	3	38%
Management of lower respiratory tract infections	-	-	3	38%
Management of lower urinary tract infections	-	-	3	38%
Management of soft tissue infections	-	-	1	13%

**Table 5:** Focus Group Recommendations for Strengthening AMS and IPC Programs in Veneto Region.

Governance and Organizational Structure
Integrate AMS and IPC objectives into General Managers’ performance targets to ensure accountability and commitment from top management. Align regional objectives with each healthcare organization’s specific needs, ensuring consistency with regional healthcare priorities through the collaboration of local health authorities in defining regional AMS and IPC plans. Establish a regional external oversight body to monitor and evaluate AMS and IPC implementation, ensuring alignment with regional and national strategies. Simplify internal governance structures to ensure clear representation of divisions and territorial entities, improving coordination and execution of AMS and IPC programs. Increase transparency by publishing organizational models, plans, and reports on organizations’ websites, making them accessible both to healthcare professionals and the general population. Develop standardized regional AMS and IPC toolkits for healthcare professionals, including indicators and minimum standards for organizational plans. Launch public campaigns via social and “traditional” media to raise awareness of the conscious use of antibiotics and IPC among the general population.
Human Resources and link professional for AMS and IPC
Set clear staffing standards for both hospitals and community care settings, ensuring adequate numbers of healthcare professionals dedicated to AMS and IPC. Expand the network of link professionals, especially in community care settings, including primary and home care. Standardize the profiles and activities of link professionals through structured training programs targeting IPC and AMS practices. Implement formal recognition systems for link professionals, offering financial incentives and continuing education credits. Strengthen collaboration between hospital-based and community-based link professionals to ensure seamless integration of AMS and IPC efforts. Formalize the annual objectives of link professionals, making them measurable and regularly monitored through performance reviews. Improve training and education programs about AMS for Primary Care Physicians.
ID consultation and AMS Support Services
Expand infectious disease consultation services in spoke hospitals and community care settings to provide continuous AMS support. Establish permanent AMS reference figures in spoke hospitals and community care facilities to provide leadership on antibiotic use. Strengthen partnerships between spoke and hub hospitals, ensuring consistent access to infectious disease expertise through shared personnel or telemedicine services. Ensure infectious disease support is available for clinical case consultations and broader AMS efforts, such as audits, policy implementation, and staff education. Fully integrate infectious disease services into daily clinical practice, making AMS a proactive component of care. Develop a telemedicine-based remote consultation system to provide timely infectious disease guidance in community settings.
Antibiotic Consumption Reports in Hospitals and Primary Care
Create a uniform regional framework for antibiotic consumption reports, utilizing consistent quantitative (DDD) and qualitative (AWaRe) indicators across healthcare settings. Encourage greater use of qualitative indicators in primary care reports, alongside training on interpretation. Promote the use of quantitative indicators like DDD in primary care alongside patient-centered metrics. Implement regional benchmarking and feedback systems to monitor antibiotic use and improve AMS interventions. Develop empiric antibiotic therapy protocols specifically for primary care to guide appropriate initial treatments. Integrate, through IT systems, antibiotic prescription data with AMR based on tools used in the veterinary field.
Other recommendations from the focus group
Develop and implement comprehensive IPC protocols for primary care and residential facilities, with a focus on infection prevention and MDRO management. Establish real-time microbiology alert systems in hospitals that communicate infection risks and MDRO presence in community care settings. Integrate microbiology alerts into shared IT platforms between hospitals and primary care to enable immediate action across all care settings. Ensure the transfer of microbiology data, including critical infection alerts, during patient discharge from hospitals to community care facilities through a coordinated IT system. Overcome obstacles imposed by Italian privacy regulation to share data between institutions, with epidemiological and decision-making for public health. Integrate data from private laboratories into AMR surveillance. Improve standardization of methods for microbiological analysis. Invest in technology to support microbiological laboratories and field epidemiology.

## Data Availability

The original contributions presented in this study are included in the article. Further inquiries can be directed to the corresponding author.

## References

[1] World Health Organization (WHO) (2023). Antimicrobial Resistance 2020. https://www.who.int/news-room/fact-sheets/detail/antimicrobial-resistance.

[2] Jesudason T. (2023). A One Health priority research agenda for AMR. Lancet Microbe.

[3] O’Neill J. (2016). Tackling Drug-Resistant Infections Globally: Final Report and Recommendations. Review on Antimicrobial Resistance.

[4] Cassini A., Högberg L.D., Plachouras D., Quattrocchi A., Hoxha A., Simonsen G.S., Colomb-Cotinat M., Kretzschmar M.E., Devleesschauwer B., Cecchini M. (2019). Attributable deaths and disability-adjusted life-years caused by infections with antibiotic-resistant bacteria in the EU and the European Economic Area in 2015: A population-level modelling analysis. Lancet Infect. Dis..

[5] Italian Medicines Agency (Agenzia Italiana del Farmaco—AIFA) (2023). The Medicines Utilisation Monitoring Centre. National Report on Medicines Use in Italy. Year 2022. https://www.aifa.gov.it/en/rapporti-osmed.

[6] World Health Organization (WHO) (2015). Global Action Plan on Antimicrobial Resistance. https://www.who.int/initiatives/sdg3-global-action-plan.

[7] Willemsen A., Reid S., Assefa Y. (2022). A review of national action plans on antimicrobial resistance: Strengths and weaknesses. Antimicrob. Resist. Infect. Control.

[8] Ministry of Health, Italy (2017). National Action Plan on Antibiotics Resistance 2017–2020. https://www.who.int/publications/m/item/italy-national-plan-against-antimicrobial-resistance.

[9] Ministry of Health, Italy (2022). National Action Plan on Antibiotics Resistance 2022–2025. https://www.salute.gov.it/imgs/C_17_pubblicazioni_3294_allegato.pdf.

[10] Bravo G., Cattani G., Malacarne F., Tricarico P., Arnoldo L., Brunelli L., Zotti C., Moro M.L., Diegoli G., Pezzotti P. (2022). SPiNCAR: A systematic model to evaluate and guide actions for tackling AMR. PLoS ONE.

[11] Ferre F., de Belvis A.G., Valerio L., Longhi S., Lazzari A., Fattore G., Ricciardi W., Maresso A. (2014). Italy: Health system review. Health Syst. Transit..

[12] Veneto Region (2016). Regional Law No 19 of October 25, 2016. Establishment of the Governance Body for Regional Healthcare in Veneto, Called “Azienda per il Governo della Sanità della Regione del Veneto—Azienda Zero”. Provisions for Defining the New Territorial Areas of the ULSS Companies. https://www.consiglioveneto.it/web/crv/dettaglio-legge?catStruttura=LR&anno=2016&numero=19&tab=storico.

[13] Mauro M., Giancotti M. (2023). The 2022 primary care reform in Italy: Improving continuity and reducing regional disparities?. Health Policy.

[14] Veneto Region (2019). Regional Decree of Veneto Region No. 1402 of October 1, 2019. “National Action Plan on Antimicrobial Resistance (PNCAR) 2017–2020”. Approval of the Documents Titled “Veneto Region Strategy for the Proper Use of Antibiotics in Human Health” and “Regional Plan for the Surveillance, Prevention, and Control of Healthcare-Associated Infections (HAIs)”. https://bur.regione.veneto.it/BurvServices/Pubblica/DettaglioDgr.aspx?id=404556.

[15] Veneto Region (2018). Regional Decree of Veneto Region No. 1912 of December 21, 2018. “Update of the Regional Committee for the Prevention and Control of Healthcare-Associated Infections, within the framework of the National Action Plan on Antimicrobial Resistance (PNCAR) 2017–2020, and the Hospital Infection Control Committee (CIO)”. https://bur.regione.veneto.it/BurvServices/pubblica/DettaglioDgr.aspx?id=418279#:~:text=1912%20del%2021%20dicembre%202018,specialista%20in%20malattie%20infettive%20all’.

[16] Dawson S.J. (2003). The role of the infection control link nurse. J. Hosp. Infect..

[17] Broom A., Kenny K., Kirby E., Davis M., Dodds S., Post J., Broom J. (2021). The modern hospital executive, micro improvements, and the rise of antimicrobial resistance. Soc. Sci. Med..

[18] Jeleff M., Haddad C., Kutalek R. (2023). Between Superimposition and Local Initiatives: Making Sense of ‘Implementation Gaps’ as a Governance Problem of Antimicrobial Resistance. SSM Qual. Res. Health.

[19] Howard P., Pulcini C., Levy Hara G., West R.M., Gould I.M., Harbarth S., Nathwani D. (2015). An international cross-sectional survey of antimicrobial stewardship programmes in hospitals. J. Antimicrob. Chemother..

[20] Vicentini C., Corcione S., Lo Moro G., Mara A., De Rosa F.G., Zotti C.M., Collaborating Group “Unità Prevenzione Rischio Infettivo (UPRI), Regione Piemonte” (2024). Impact of COVID-19 on antimicrobial stewardship activities in Italy: A region-wide assessment. Antimicrob. Resist. Infect. Control.

[21] Vicentini C., Blengini V., Libero G., Martella M., Zotti C.M., Working Group “Unità Prevenzione Rischio Infettivo (UPRI), Regione Piemonte” (2022). Tailoring Antimicrobial Stewardship (AMS) Interventions to the Cultural Context: An Investigation of AMS Programs Operating in Northern Italian Acute-Care Hospitals. Antibiotics.

[22] Ashiru-Oredope D., Budd E.L., Bhattacharya A., Din N., McNulty C.A., Micallef C., Ladenheim D., Beech E., Murdan S., Hopkins S. (2016). Implementation of antimicrobial stewardship interventions recommended by national toolkits in primary and secondary healthcare sectors in England: TARGET and Start Smart Then Focus. J. Antimicrob. Chemother..

[23] Mathew P., Sivaraman S., Chandy S. (2019). Communication strategies for improving public awareness on appropriate antibiotic use: Bridging a vital gap for action on antibiotic resistance. J. Fam. Med. Prim. Care.

[24] Società Scientifica Nazionale Infermieri Specialisti Rischio Infettivo (ANIPIO) (2021). Le Infezioni Correlate all’Assistenza (ICA): Una Pandemia Silente.

[25] Dickstein Y., Nir-Paz R., Pulcini C., Cookson B., Beović B., Tacconelli E., Nathwani D., Vatcheva-Dobrevska R., Rodríguez-Baño J., Hell M. (2016). Staffing for infectious diseases, clinical microbiology and infection control in hospitals in 2015: Results of an ESCMID member survey. Clin. Microbiol. Infect..

[26] Italian Ministry of Health (1988). Circular of the Ministry of Health No. 8/1988: Fight against hospital infections: Surveillance.

[27] European Centre for Disease Prevention and Control (2024). Point Prevalence Survey of Healthcare Associated Infections and Antimicrobial Use in European Acute Care Hospitals. https://www.ecdc.europa.eu/en/publications-data/PPS-HAI-AMR-acute-care-europe-2022-2023.

[28] Dellit T.H., Owens R.C., McGowan J.E., Gerding D.N., Weinstein R.A., Burke J.P., Huskins W.C., Paterson D.L., Fishman N.O., Carpenter C.F. (2007). Infectious Diseases Society of America and the Society for Healthcare Epidemiology of America guidelines for developing an institutional program to enhance antimicrobial stewardship. Clin. Infect. Dis..

[29] Ciccarello C., Leber M.B., Leonard M.C., Nesbit T., Petrovskis M.G., Pherson E., Pillen H.A., Proctor C., Reddan J. (2021). ASHP Guidelines on the Pharmacy and Therapeutics Committee and the Formulary System. Am. J. Health Syst. Pharm..

[30] Polidori P., Leonardi Vinci D., Adami S., Bianchi S., Faggiano M.E., Provenzani A. (2022). Role of the hospital pharmacist in an Italian antimicrobial stewardship programme. Eur. J. Hosp. Pharm..

[31] Burnham J.P., Fritz S.A., Yaeger L.H., Colditz G.A. (2019). Telemedicine Infectious Diseases Consultations and Clinical Outcomes: A Systematic Review. Open Forum Infect. Dis..

[32] Mailig M., Cookson N.A., Schulz L.T. (2022). Telestewardship programs support clinical care and improve fiscal outcomes across the continuum through partnership between hospitals and health systems: A systematic review. Am. J. Health Syst. Pharm..

[33] Klatt M.E., Schulz L.T., Fleischman D., Fox B.C., Burke S., Grinder D., Rose W.E., Lepak A.J., Andes D.R. (2021). Implementation of telehealth antimicrobial stewardship through partnership of an academic medical center and a community hospital. Am. J. Health Syst. Pharm..

[34] Vento T.J., Veillette J.J., Gelman S.S., Adams A., Jones P., Repko K., Stenehjem E.A. (2021). Implementation of an Infectious Diseases Telehealth Consultation and Antibiotic Stewardship Program for 16 Small Community Hospitals. Open Forum Infect. Dis..

[35] Vestesson E., De Corte K., Chappell P., Crellin E., Clarke G.M. (2023). Antibiotic prescribing in remote versus face-to-face consultations for acute respiratory infections in primary care in England: An observational study using target maximum likelihood estimation. EClinicalMedicine.

[36] Ferrari E., Scannavini P., Palandri L., Fabbri E., Tura G., Bedosti C., Zanni A., Mosci D., Righi E., Vecchi E. (2025). Training in infection prevention and control: Survey on the volume and on the learning demands of healthcare-associated infections control figures in the Emilia-Romagna Region (Northern Italy). Ann. Ig. Med. Prev. Comunità.

[37] Saha S.K., Thursky K., Kong D.C.M., Mazza D. (2022). A Novel GPPAS Model: Guiding the Implementation of Antimicrobial Stewardship in Primary Care Utilising Collaboration between General Practitioners and Community Pharmacists. Antibiotics.

[38] Del Fabro G., Venturini S., Avolio M., Basaglia G., Callegari A., Bramuzzo I., Basso B., Zanusso C., Rizzo A., Tonutti G. (2024). Time is running out. No excuses to delay implementation of antimicrobial stewardship programmes: Impact, sustainability, resilience and efficiency through an interrupted time series analysis (2017–2022). JAC Antimicrob. Resist..

[39] Dal Bò O., Fabro R., Faruzzo A., Tignonsini D., Malacarne F., Cocconi R. (2017). Link Professional: Training Project for Reduction of healthcare-associated infections. Gimpios.

[40] Birgand G., Castro-Sánchez E., Hansen S., Gastmeier P., Lucet J.C., Ferlie E., Holmes A., Ahmad R. (2018). Comparison of governance approaches for the control of antimicrobial resistance: Analysis of three European countries. Antimicrob. Resist. Infect. Control.

[41] Regulation (EU) 2016/679 of the European Parliament and of the Council of 27 April 2016 on the Protection of Natural Persons with Regard to the Processing of Personal Data and on the Free Movement of Such Data, and Repealing Directive 95/46/EC (General Data Protection Regulation). https://eur-lex.europa.eu/eli/reg/2016/679/oj.

[42] Società Italiana di Leadership e Management in Medicina and Istituto Italiano per la Privacy e la Valorizzazione dei Dati—Consensus Statement (2024). Proposta di Revisione della Normativa Privacy in Sanità. https://www.medici-manager.it/wp-content/uploads/2024/02/Privacy-in-10-punti-la-proposta-di-SIMM_last_REV.pdf.

[43] Broom J., Broom A., Kirby E., Gibson A.F., Post J.J. (2017). Individual care versus broader public health: A qualitative study of hospital doctors’ antibiotic decisions. Infect. Dis. Health.

[44] Vukovic J., Ivankovic D., Habl C., Dimnjakovic J. (2022). Enablers and barriers to the secondary use of health data in Europe: General data protection regulation perspective. Arch. Public Health.

